# Prognostic Impact of New-Onset Type 2 Diabetes Mellitus After Acute Myocardial Infarction: Long-Term Mortality Compared with Pre-Existing and No Diabetes

**DOI:** 10.3390/medicina62030430

**Published:** 2026-02-25

**Authors:** Ygal Plakht, Tamara Yakubov, Harel Gilutz, Keren Skalsky, Alon Shechter, Shani Dahan, Arthur Shiyovich

**Affiliations:** 1 Faculty of Health Sciences, Ben-Gurion University of the Negev, Beer Sheva 8410501, Israel; plakht@bgu.ac.il (Y.P.); gilutz@bgu.ac.il (H.G.);; 2 Emergency Medicine Department, Soroka University Medical Center, Beer Sheva 8410101, Israel; 3 Internal Medicine Department, Soroka University Medical Center, Beer Sheva 8410101, Israel; yakubovtamara@gmail.com; 4 Department of Cardiology, Rabin Medical Center, Tel Aviv University, Petach Tikva 4910000, Israel; 5 Division of Cardiology, Assuta Ashdod Medical Center, Ashdod 7747629, Israel; 6 Division of Cardiology, Massachusetts General Hospital, Harvard Medical School, Boston, MA 02115, USA; 7 Heart and Vascular Institute, Brigham and Women’s Hospital, Harvard Medical School, 75 Francis Street, Boston, MA 02115, USA

**Keywords:** acute myocardial infarction, new-onset diabetes mellitus, long-term mortality

## Abstract

*Background and Objectives*: Acute myocardial infarction (AMI) increases the risk of developing type 2 diabetes mellitus (T2DM), yet the long-term prognostic significance of new-onset T2DM after AMI remains unclear. We aimed to evaluate long-term mortality among AMI survivors who developed T2DM during follow-up compared with those who remained non-diabetic and those with pre-existing T2DM. *Materials and Methods*: We conducted a retrospective cohort study of consecutive AMI patients hospitalized between 2002 and 2017 at a large tertiary medical center. Patients were categorized into three groups: (1) new-onset T2DM after AMI (NOT2DM), 1–15 years post-discharge; (2) no diabetes (No DM), with no evidence of T2DM during the same period; (3) pre-existing T2DM (T2DM), diagnosed prior to or during the index AMI. Age- and sex-matching was performed. Primary outcome: all-cause mortality with up-to 10 years of follow-up. *Results*: A total of 4207 patients were included (1202 NOT2DM; 2404 No DM; 601 T2DM). Over a median follow-up of 2729 days, 1492 patients (35.5%) died. Mortality was highest in the NOT2DM group (44.9%) compared with No DM (31.2%) and T2DM (33.4%) (*p* < 0.001). After adjustment, NOT2DM remained strongly associated with increased mortality versus No DM (AdjHR 1.408; 95% CI 1.256–1.578) and T2DM (AdjHR 1.272; 95% CI 1.074–1.508) (*p* < 0.001 for each). The mortality impact was most pronounced in patients < 65 years. *Conclusions*: Development of T2DM after AMI identifies a high-risk subgroup with significantly worse long-term survival, especially among the young patients. These findings underscore the need for systematic metabolic surveillance and early preventive strategies in AMI survivors.

## 1. Introduction

Type 2 diabetes mellitus (T2DM) is a major global contributor to atherosclerotic cardiovascular disease (ASCVD), with its prevalence and clinical burden continuing to rise worldwide [1,2,3]. Coronary artery disease (CAD) remains the leading cause of morbidity and mortality among individuals with diabetes [1,4]. Emerging evidence shows that cardiovascular risk begins long before diabetes is diagnosed. Studies have demonstrated an increased risk of developing T2DM after an acute myocardial infarction (AMI) [5,6,7]. Moreover, individuals who eventually develop T2DM exhibit approximately a higher incidence of AMI and ischemic stroke for up to three decades before the clinical diagnosis [8].

AMI and T2DM share overlapping cardiometabolic risk factors, including insulin resistance, visceral adiposity, dyslipidemia, and inflammation. Beyond this overlap, AMI itself appears to accelerate the development of new-onset diabetes mellitus (NOT2DM). Systemic inflammatory activation after AMI, together with neurohormonal changes and reduced insulin sensitivity, may promote diabetes onset [5,7,9,10]. The incidence of NOT2DM following AMI is significant, affecting more than half of patients [5].

Despite growing evidence describing which patients develop T2DM after AMI, the prognostic implications of this transition remain poorly defined. In particular, it is not well established whether AMI survivors who develop T2DM during follow-up experience different long-term outcomes compared with those who remain non-diabetic or those with pre-existing diabetes at the time of the infarction.

The objective of this study was to evaluate long-term mortality among AMI survivors who develop T2DM after the index event, compared with survivors without diabetes and those with established diabetes before AMI.

## 2. Methods

### 2.1. Study Population and Setting

This retrospective cohort study utilized data from the SAMI-III (Soroka Acute Myocardial Infarction) registry, which captures all patients hospitalized with AMI at Soroka University Medical Center (SUMC) between 2002 and 2017 [5]. For individuals with multiple AMI admissions during the study period, the first hospitalization was designated as the index event.

Patients were excluded if they were non-Israeli citizens; died during the index hospitalization or within one year after discharge; had a history of type 1 diabetes mellitus; received a diagnosis of T2DM within one year after discharge; or had insufficient information to determine T2DM status (no recorded diagnosis and no hemoglobin A1c [HbA1c] or blood glucose measurements from one year prior to admission through 15 years post-discharge) [5].

### 2.2. Study Groups

Eligible patients were categorized into three mutually exclusive groups.

#### 2.2.1. New-Onset T2DM After AMI (NOT2DM)

For patients who developed T2DM 1–15 years after discharge, NOT2DM was defined by the following criteria: the registration of a T2DM diagnosis (by the International Classification of Diseases, Ninth Revision, Clinical Modification [ICD-9-CM] codes 250) or laboratory results meeting the 2023 criteria of the American Diabetes Association (ADA) [11]. According to this definition, any patients with two HbA1c test results of ≥6.5% or two random blood glucose test results of ≥200 mg/dL were classified as having NOT2DM. The index date for this group was the date of first documented T2DM diagnosis.

#### 2.2.2. No Diabetes (No DM)

Patients with no T2DM diagnosis during the same 1–15-year post-discharge window. Group matching (2:1 ratio to NOT2DM) was performed by age (within a window of 10 years) and sex. For comparability, an assigned index date was generated within the same 1–15-year window as for NOT2DM using matched age–sex strata.

#### 2.2.3. Pre-Existing T2DM (T2DM)

Patients with documented T2DM (applying same diagnostic criteria as above mentioned) before or during the AMI hospitalization. Matching to the NOT2DM group was performed in the same manner, at a 0.5:1 ratio, due to limited sample size in some strata. Similarly, the assigned index date was matched to the NOT2DM group.

### 2.3. Outcome

The primary outcome was all-cause mortality from the index date. Follow-up continued for up to 10 years (maximum 3652 days) or until 31 July 2023, whichever occurred first.

### 2.4. Covariates and Definitions

Baseline characteristics were extracted from the computerized medical records of SUMC database. As in prior analyses from this registry, available data included demographic information, cardiovascular risk factors, comorbid conditions, laboratory values, echocardiographic and coronary angiographic findings, and details of AMI management. Follow-up information was obtained from SUMC electronic records and from affiliated community clinics [5,12]. Comorbidities were identified using ICD-9-CM codes documented by the treating clinicians.

The index hospitalization was defined as the first admission with a diagnosis of AMI, established by ischemic symptoms and/or electrocardiographic changes together with a characteristic rise and fall in cardiac biomarkers consistent with acute myocardial injury (ICD-9-CM 410): ST-elevation AMI (STEMI) (410.0–410.6) and Non-ST-elevation AMI (NSTEMI) (410.7–410.9). Dyslipidemia was defined by a low-density lipoprotein (LDL) cholesterol level ≥ 100 mg/dL at any point during the 12 months before or after the index event [13]. Obesity was defined as a body mass index (BMI) ≥ 30 kg/m^2^ and anemia as hemoglobin <13 g/dL in men or <12 g/dL in women. Significant coronary artery disease was defined as ≥70% luminal stenosis on coronary angiography. Severe left ventricular dysfunction was defined as an ejection fraction < 30% on the initial in-hospital echocardiogram, whereas pulmonary hypertension was defined as an estimated pulmonary artery systolic pressure ≥ 37 mmHg on the same study. Valvular heart disease (mitral or tricuspid regurgitation) was considered clinically significant if graded as moderate or greater by experienced echocardiographers in accordance with American Society of Echocardiography guidelines [14].

### 2.5. Statistical Analysis

Statistical analysis was performed using the Statistical Package for the Social Sciences (SPSS), version 29 (IBM Corporation, Armonk, NY, USA). Variables were reported as frequencies and percentages, medians and interquartile ranges (IQRs), or means and standard deviations (SDs) and compared using Pearson’s chi-square supplemented by Bonferroni-adjusted z-tests for post hoc column-proportion comparisons, and analysis of variance (ANOVA) followed by Tukey’s HSD post hoc testing.

The time-dependent probability and cumulative mortality were assessed using the Kaplan–Meier survival approach and compared using the Log-rank test adjusted with Bonferroni correction. Independent associations with the risk of all-cause death were evaluated using Cox proportional hazard regression analyses at univariable and multivariable levels. In these models, the group of No DM served as the reference group. In addition, the risks of mortality for the NOT2DM group were compared with those for individuals with pre-existing T2DM. Multivariable analysis incorporated baseline variables demonstrating a *p*-value of <0.1 at the univariable stage using a stepwise approach. Lastly, a sub-group analysis was undertaken, stratifying the cohort by predefined age groups. Missing data for select clinical variables, such as echocardiographic and coronary angiographic findings, were handled by assigning a distinct “missing” category within each variable. The results of the models were presented as hazard ratios (HR)/adjusted hazard ratios (AdjHR) with their 95% confidence intervals (CI). Statistical significance required a two-sided *p*-value of <0.05.

## 3. Results

### 3.1. Cohort Selection and Baseline Characteristics

**Figure 1:**
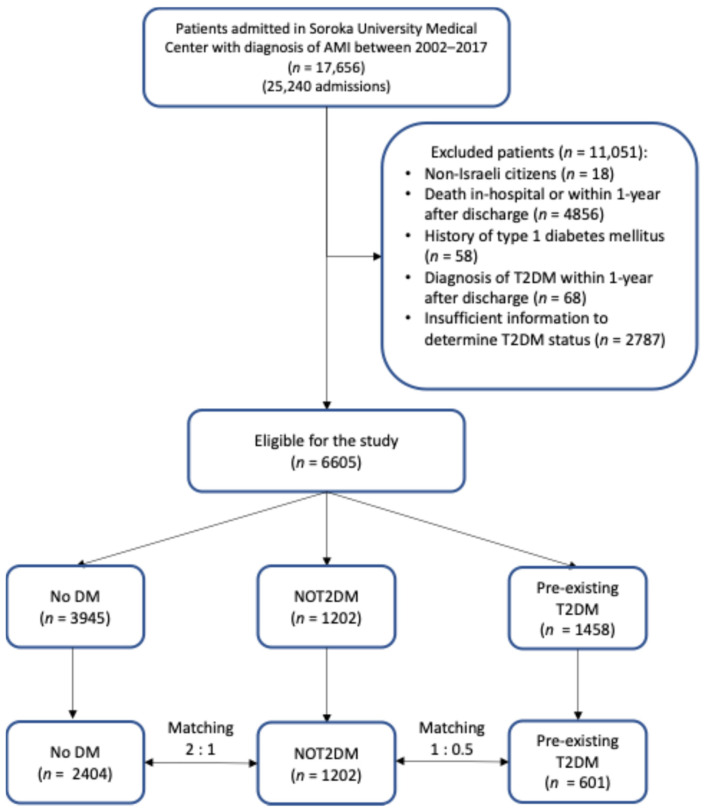
Study flowchart. AMI: acute myocardial infarction; No DM: no diabetes mellitus; NOT2DM: new-onset type 2 diabetes mellitus after AMI; T2DM: type 2 diabetes mellitus.

Before matching, the corresponding group sizes were 1202 (NOT2DM), 3945 (No DM), and 1458 (T2DM). After applying the inclusion and exclusion criteria (Figure 1), a total of 4207 patients were included in the analysis: 1202 in the NOT2DM group, 2404 in the No DM group, and 601 in the pre-existing T2DM group. Mean age was 62 years, 76% men.

Baseline characteristics are summarized in Table 1. Compared with the No DM group, patients in the NOT2DM group had a higher representation of minority populations and a greater prevalence of several cardiovascular and metabolic risk factors, including prior myocardial infarction, hypertension, obesity, peripheral vascular disease, and pulmonary hypertension. They were also less likely to present with STEMI, be admitted to the intensive cardiac care unit, or undergo invasive management during the index hospitalization.

In comparison with the T2DM group, the NOT2DM cohort exhibited higher rates of prior MI and STEMI but lower rates of congestive heart failure, chronic kidney disease, hypertension, and left ventricular hypertrophy. HbA1c values obtained around the time of the index AMI demonstrated that prediabetes (HbA1c 5.7–6.5%) was more common among NOT2DM patients than among those in the No DM group.

### 3.2. Follow-Up and Mortality

**Figure 2:**
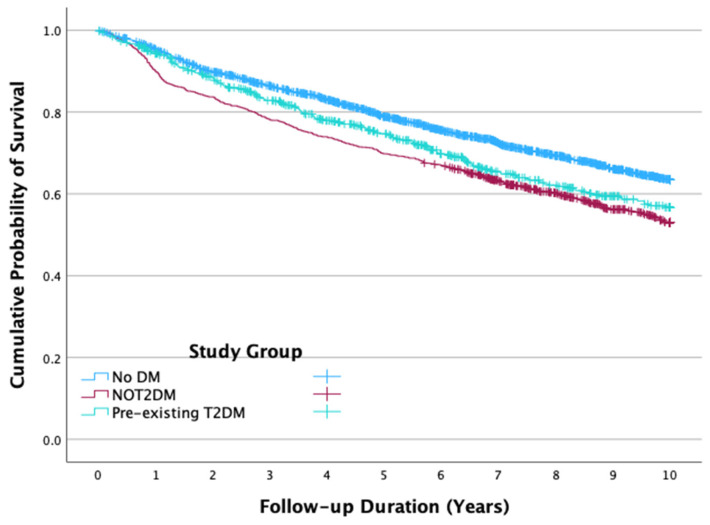
Cumulative survival functions for post-acute myocardial infarction all-cause mortality throughout the follow-up period up to 10 years, by study group. No DM: no diabetes mellitus; NOT2DM: new-onset type 2 diabetes mellitus after AMI; T2DM: type 2 diabetes mellitus.

The median follow-up duration was 2729 days (IQR 1365–3652). During this period, 1492 patients (35.5%) died. Mortality rates differed significantly across groups: 44.9% in the NOT2DM group, 31.2% in the No DM group, and 33.4% in the T2DM group (*p* < 0.001). Cumulative mortality was highest among patients who developed NOT2DM (0.470), significantly greater than in the No DM cohort (0.365, *p* < 0.001) and numerically higher than in the T2DM group (0.433, *p* = 0.097) (Figure 2).

### 3.3. Risk of Mortality

In univariable analyses, both NOT2DM and pre-existing T2DM were associated with higher mortality relative to the No DM group, with the HRs of 1.432 (95% CI: 1.282–1.599; *p* < 0.001) for NOT2DM and 1.258 (95% CI: 1.077–1.471; *p* = 0.004) for T2DM. The NOT2DM group also showed a nonsignificant trend toward higher mortality compared with the T2DM group (HR = 1.138; 95% CI: 0.968–1.339; *p* = 0.118).

In multivariable Cox model (Table 2), the association between NOT2DM and increased mortality persisted. NOT2DM had the highest mortality risk, with a 41% higher adjusted risk of death compared with the No DM group (AdjHR 1.408; 95% CI: 1.256–1.578; *p* < 0.001). Pre-existing T2DM was not associated with a statistically significant increase in adjusted mortality relative to No DM (AdjHR 1.106; 95% CI: 0.942–1.299; *p* = 0.218). Furthermore, NOT2DM also had a significantly higher mortality risk than pre-existing T2DM (AdjHR 1.272; 95% CI: 1.074–1.508; *p* = 0.005).

### 3.4. Subgroup Analysis

**Figure 3:**
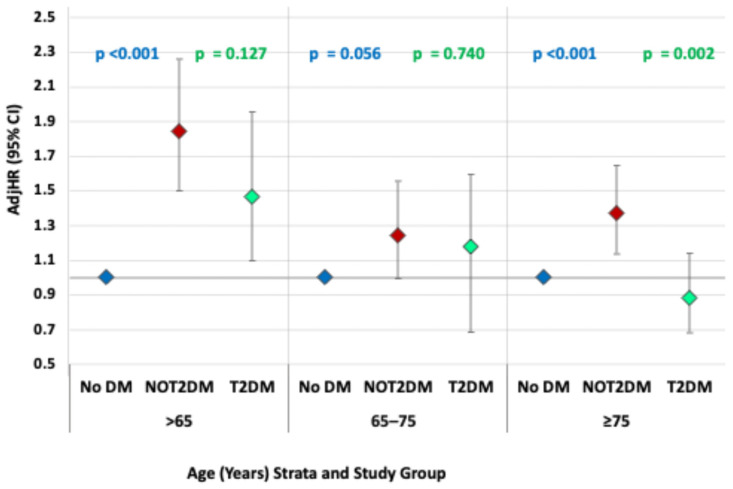
Association between type 2 diabetes mellitus status and post–acute myocardial infarction all-cause mortality up to 10 years of follow-up, stratified by age groups, based on multivariable analyses (see also Table 3). AMI: acute myocardial infarction; No DM: no diabetes mellitus; NOT2DM: new-onset type 2 diabetes mellitus after AMI; T2DM: pre-existing type 2 diabetes mellitus; AdjHR: adjusted hazard ratio; CI: confidence interval. AdjHRs for all-cause mortality following AMI, stratified by age groups and diabetes status (multivariable analyses). The group of No DM was used as the reference group for each age strata. Blue *p*-values indicate comparisons between No DM and NOT2DM, while green *p*-values indicate comparisons between NOT2DM and pre-existing T2DM.

Across all age strata, patients with NOT2DM exhibited consistently higher mortality risk compared with the No DM group, with borderline statistical significance in the 65–75 year category (*p* = 0.056). In contrast, the association between pre-existing T2DM and mortality progressively weakened with advancing age and was statistically significant only among patients younger than 65 years. In the oldest age group, NOT2DM was associated with a significantly higher risk of death compared with pre-existing T2DM. These findings are illustrated in Figure 3 and detailed in Table 3.

## 4. Discussion

In this large cohort of AMI survivors, development of NOT2DM after AMI was associated with significantly worse long-term prognosis than both patients who remained non-diabetic and those who had pre-existing T2DM at the time of AMI. Specifically, NOT2DM patients exhibited a ~40% higher adjusted mortality risk compared with the non-diabetic group and ~27% higher risk compared with the pre-existing T2DM cohort. The prognostic impact was particularly pronounced in patients younger than 65 years.

Multiple studies have shown that a substantial proportion of individuals already have established cardiovascular disease at the time T2DM is first diagnosed [15,16,17]. Extending this concept, Gyldenkerne et al. [8] demonstrated that individuals who go on to develop T2DM exhibit a markedly elevated burden of cardiovascular events decades before clinical onset. In their nationwide registry analysis, the incidence of MI, ischemic stroke, and other major cardiovascular events was nearly two-fold higher beginning 20–30 years prior to diabetes diagnosis, and this excess risk persisted thereafter.

It is also well established that the presence of T2DM at the time of AMI confers a significantly worse prognosis [18,19]. Finally, we and others have shown that survivors of AMI are themselves at increased risk of developing T2DM during follow-up. This heightened risk reflects a greater burden of shared cardiometabolic comorbidities—such as obesity, dyslipidemia, and hypertension—as well as the metabolic consequences of AMI and its treatments [5,7,9,10].

However, the literature remains sparse regarding the long-term prognostic implications of incident T2DM following AMI. Our findings add novel insights by demonstrating that patients who develop new-onset diabetes after AMI (NOT2DM) represent a distinct high-risk subgroup with worse long-term outcomes, potentially even compared with those with known diabetes at baseline. Several pathophysiologic mechanisms may help explain our findings. First, the acute inflammatory and metabolic disturbances accompanying AMI may accelerate underlying β-cell dysfunction and worsen insulin resistance, thereby unmasking latent dysglycemia in predisposed individuals. AMI induces a systemic inflammatory response and neurohormonal activation, including catecholamine and cortisol surges, which can impair glucose regulation for weeks to months. Association and potential interaction between T2DM and other cardiovascular risk factors in determining prognosis. Reduced physical activity during recovery and post-AMI weight gain may further worsen insulin sensitivity, collectively facilitating the transition from prediabetes to overt T2DM [1,5,7,9,10,20].

Second, patients who eventually developed NOT2DM in our cohort appeared to carry a higher cardiometabolic burden at baseline—including more frequent prior MI, hypertension, obesity, peripheral vascular disease, and pulmonary hypertension. In a recent analysis from the same registry, cardiomegaly, prior MI, atrioventricular block, hypertension, smoking, peripheral vascular disease, obesity, NSTEMI (vs. STEMI), mitral regurgitation, dyslipidemia, and elevated HbA1c were independently associated with incident NOT2DM [5]. This suggests that NOT2DM may function as a marker of a more aggressive, yet initially unrecognized, metabolic phenotype. Many of these individuals likely had long-standing dysglycemia that remained undiagnosed at the time of AMI, resulting in delayed initiation of preventive therapies that are routinely prescribed to patients with known T2DM.

Differences in the prognostic impact of diabetes across age groups—particularly the greater relative risk observed among younger patients with diabetes presenting with AMI—have been reported previously [2,21]. Notably, our findings parallel these earlier observations, but specifically in the setting of NOT2DM emerging after AMI. While further research is needed to elucidate the underlying mechanisms, potential contributors may include differences in sex distribution, genetic susceptibility, diabetes severity, management and treatment adherence, variability in metabolic profiles, and interactions with other cardiovascular risk factors [1,2,22,23,24].

Taken together, these factors imply that the development of NOT2DM after AMI is not simply a new diagnosis but rather the clinical manifestation of a long-developing metabolic disease process that was amplified by the physiological stress of MI. This may explain why NOT2DM patients experienced worse long-term outcomes than both non-diabetic patients and those with established T2DM, whose metabolic risk was already recognized and treated at the time of the index event.

### 4.1. Clinical Implications

These findings have several important clinical implications. Post-AMI management should incorporate structured, longitudinal metabolic surveillance. Patients at elevated risk for incident diabetes—particularly younger individuals and those with prediabetic HbA1c levels or multiple metabolic comorbidities—may benefit from earlier and more intensive preventive strategies. Beyond standard secondary prevention after AMI, consideration could be given to therapies with proven metabolic and cardiovascular benefit (e.g., GLP-1 receptor agonists, SGLT2 inhibitors, or more aggressive lipid and blood pressure control) even before overt diabetes is diagnosed, although prospective data are needed to define optimal timing and patient selection.

### 4.2. Limitations

As an observational, registry-based analysis, residual confounding cannot be excluded despite extensive adjustment and matching. Although diabetes status was defined using both laboratory values and ICD codes, some degree of misclassification remains possible particularly with respect to distinguishing truly incident diabetes from previously undiagnosed disease. Data on newer cardiometabolic therapies (e.g., GLP-1 receptor agonists and SGLT2 inhibitors) were unavailable and may have influenced long-term outcomes. Additionally, cause-specific mortality could not be assessed, limiting mechanistic interpretation. The single-center design may restrict generalizability, although the large cohort and extended follow-up strengthen the findings. Furthermore, post-AMI management strategies and their intensity over time were not available in this registry, limiting our ability to account for treatment differences that could bias comparisons between patients with pre-existing T2DM and those with NOT2DM.

We were also unable to determine diabetes duration among patients with pre-existing T2DM, which may have heterogeneous prognostic implications. In addition, adjustment for pre-AMI HbA1c was not performed due to its limited availability and related methodological constraints. Finally, baseline clinical characteristics were recorded at the time of the index AMI rather than at the assigned index date, resulting in potentially substantial intervals—up to 15 years—between the AMI event and the beginning of follow-up for some individuals.

## 5. Conclusions

In survivors of AMI, the development of NOT2DM during follow-up identifies a subgroup at substantially increased long-term mortality risk—higher than both non-diabetic individuals and those with pre-existing T2DM. These findings highlight the need for proactive metabolic surveillance and early intervention strategies in AMI patients to better identify and treat emerging diabetes and improve long-term outcomes.

## Figures and Tables

**Table 1:** Baseline characteristics of the study population by study group.

Parameter	Value	Study Group			Total	*p*
		No DM	NOT2DM	T2DM		
*n*		2404	1202	601	4207	
Demographics						
Age, Years	Min–Max	23–101	25–100	32–95	23–101	
	Mean (SD)	61.91 (13.41)	62.04 (12.84)	62.41 (12.53)	62.02 (13.12)	0.697
	<65	1467 (61.0)	741 (61.6)	362 (60.2)	2570 (61.1)	0.969
	65–75	494 (20.5)	239 (19.9)	128 (21.3)	861 (20.5)	
	≥75	443 (18.4)	222 (18.5)	111 (18.5)	776 (18.4)	
Sex	Male	1818 (75.6)	909 (75.6)	455 (75.7)	3182 (75.6)	0.999
Ethnicity	Minorities	372 (15.5)	232 (19.3) †	100 (16.6)	704 (16.7)	0.015
Cardiac diseases						
Supraventricular arrhythmias		264 (11.0)	158 (13.1)	60 (10.0)	482 (11.5)	0.074
CHF		195 (8.1)	112 (9.3) ‡	80 (13.3)	387 (9.2)	<0.001
Pulmonary heart disease		93 (3.9)	62 (5.2)	44 (7.3)	199 (4.7)	0.001
CIHD		2012 (83.7)	968 (80.5)	511 (85.0)	3491 (83)	0.021
s/p MI		234 (9.7)	167 (13.9) †‡	56 (9.3)	457 (10.9)	<0.001
s/p PCI		246 (10.2)	127 (10.6) ‡	93 (15.5)	466 (11.1)	<0.001
s/p CABG		124 (5.2)	78 (6.5)	48 (8.0)	250 (5.9)	0.020
AV block		67 (2.8)	42 (3.5)	18 (3.0)	127 (3.0)	0.504
Cardiovascular risk factors						
Chronic kidney disease		81 (3.4)	41 (3.4) ‡	37 (6.2)	159 (3.8)	0.004
Dyslipidemia		1727 (71.8)	905 (75.3)	480 (79.9)	3112 (74.0)	<0.001
Hypertension		1043 (43.4)	596 (49.6) †‡	366 (60.9)	2005 (47.7)	<0.001
Obesity		482 (20.0)	329 (27.4) †	178 (29.6)	989 (23.5)	<0.001
Smoking		1197 (49.8)	639 (53.2)	274 (45.6)	2110 (50.2)	0.009
PVD		140 (5.8)	115 (9.6) †	50 (8.3)	305 (7.2)	<0.001
Family history of IHD		285 (11.9)	98 (8.2) †	69 (11.5)	452 (10.7)	0.003
Other disorders						
COPD		119 (5.0)	79 (6.6)	28 (4.7)	226 (5.4)	0.088
Neurological disorders		207 (8.6)	106 (8.8)	73 (12.1)	386 (9.2)	0.024
Malignancy		60 (2.5)	21 (1.7)	15 (2.5)	96 (2.3)	0.340
Anemia		850 (35.4)	381 (31.7)	215 (35.8)	1446 (34.4)	0.068
Schizophrenia/Psychosis		29 (1.2)	15 (1.2)	3 (0.5)	47 (1.1)	0.296
Alcohol/drug addiction		56 (2.3)	21 (1.7)	6 (1.0)	83 (2.0)	0.089
History of malignancy		103 (4.3)	52 (4.3)	30 (5.0)	185 (4.4)	0.744
Characteristics of AMI						
Year of admission	2002–2007	1225 (51.0)	806 (67.1) †‡	210 (34.9)	2241 (53.3)	<0.001
	2008–2012	813 (33.8)	311 (25.9) †	182 (30.3)	1306 (31.0)	
	2013–2017	366 (15.2)	85 (7.1) †‡	209 (34.8)	660 (15.7)	
Type of AMI	STEMI	1426 (59.3)	637 (53.0) †‡	270 (44.9)	2333 (55.5)	<0.001
Admitted/transposed to ICCU		1968 (81.9)	920 (76.5) †	456 (75.9)	3344 (79.5)	<0.001
Length of hospital stay, days	Mean (SD)	9.01 (6.29)	9.29 (7.76)	9.06 (6.58)	9.09 (6.78)	0.500
	≥7	1065 (44.3)	545 (45.3)	269 (44.8)	1879 (44.7)	0.838
Type of treatment	Noninvasive	349 (14.5)	245 (20.4) †	99 (16.5)	693 (16.5)	<0.001
	PCI	1731 (72.0)	812 (67.6) †	421 (70.0)	2964 (70.5)	
	CABG	324 (13.5)	145 (12.1)	81 (13.5)	550 (13.1)	
Acute in-hospital events						
Cardiac arrest		1 (0)	3 (0.2)	0	4 (0.1)	0.116
Cardiogenic shock		16 (0.7)	6 (0.5)	9 (1.5)	31 (0.7)	0.054
Intra-aortic balloon pulsation		60 (2.5)	26 (2.2)	8 (1.3)	94 (2.2)	0.220
Any form of pacing		40 (1.7)	28 (2.3)	8 (1.3)	76 (1.8)	0.235
Mechanical ventilation		46 (1.9)	24 (2.0)	10 (1.7)	80 (1.9)	0.886
Blood transfusion		203 (8.4)	90 (7.5)	49 (8.2)	342 (8.1)	0.612
Results of echocardiography						
Echocardiography performance	*n*	2135	1026	508	3669	
Severe LV dysfunction		133 (6.2)	65 (6.3)	44 (8.7)	242 (6.6)	0.129
LV hypertrophy		63 (3.0)	41 (4.0) ‡	37 (7.3)	141 (3.8)	<0.001
Mitral regurgitation		66 (3.1)	47 (4.6)	22 (4.3)	135 (3.7)	0.080
Tricuspid regurgitation		32 (1.5)	24 (2.3)	12 (2.4)	68 (1.9)	0.171
Pulmonary hypertension		61 (2.9)	52 (5.1) †	31 (6.1)	144 (3.9)	<0.001
Results of angiography						
Angiography performance	*n*	2003	935	470	3408	
Measure of CAD	No/non-significant	86 (4.3)	37 (4.0)	25 (5.3)	148 (4.3)	0.035
	One vessel	650 (32.5)	281 (30.1)	121 (25.7)	1052 (30.9)	
	Two vessels	600 (30.0)	270 (28.9)	136 (28.9)	1006 (29.5)	
	Three vessels/LM	667 (33.3)	347 (37.1)	188 (40.0)	1202 (35.3)	
HbA1C, %	*n*	498	211	316	1025	
	Mean (SD)	5.67 (0.40)	5.89 (0.35) †‡	6.31 (0.86)	5.92 (0.64)	<0.001
	<5.7	272 (54.6)	70 (33.2) †	75 (23.7)	417 (43.7)	<0.001
	5.7–6.5	226 (45.4)	141 (66.8) †‡	134 (42.4)	501 (48.9)	
	6.5–7.0			67 (21.2)		
	≥7.0			40 (12.7)		
Insulin treatment				38 (6.3)		
Diabetic neuropathy				8 (1.3)		
Diabetic retinopathy				12 (2.0)		
Diabetic vasculopathy				32 (5.3)		
Diabetic nephropathy				12 (2.0)		
Non complicated T2DM				548 (91.2)		
Period from discharge to index date, years	Min–Max	1–15	1–15	1–15	1–15	
	Median [IQR]	4.0 [2.0–7.0]	4.0 [2.0–7.0]	4.0 [2.0–7.0]	4.0 [2.0–7.0]	
	Mean (SD)	4.98 (3.21)	4.98 (3.21)	4.98 (3.21)	4.98 (3.21)	1.000
Age at index date, years	Min–Max	26–103	31–102	35–98	26–103	
	Mean (SD)	66.89 (13.1)	67.01 (12.54)	67.39 (12.45)	67.00 (12.85)	0.689
	<65	1135 (47.2)	583 (48.5)	278 (46.3)	1196 (47.4)	0.831
	65–75	608 (25.3)	291 (24.2)	160 (26.6)	1059 (25.2)	
	≥75	661 (27.5)	328 (27.3)	163 (27.1)	1152 (27.4)	

† Comparison between NOT2DM and No DM, ‡ Comparison between NOT2DM and T2DM. AMI: acute myocardial infarction; AV block: atrioventricular block; CABG: coronary artery bypass grafting; CAD: coronary artery disease; CHF: congestive heart failure; CIHD: chronic ischemic heart disease; COPD: chronic obstructive pulmonary disease; HbA1c: hemoglobin A1c; ICCU: intensive coronary care unit; IHD: ischemic heart disease; IQR: interquartile range; LM: left main; LV: left ventricular; MI: myocardial infarction; No DM: no diabetes mellitus; NOT2DM: new-onset type 2 diabetes mellitus after AMI; PCI: percutaneous coronary intervention; PVD: peripheral vascular disease; SD: standard deviation; s/p: status post; STEMI: ST-elevation myocardial infarction; T2DM: type 2 diabetes mellitus.

**Table 2:** Relationships between the status of type 2 diabetes mellitus and the risk for post-acute myocardial infarction all-cause mortality throughout the follow-up period up to 10 years—multivariable analysis.

Parameter	Value	B (SE)	AdjHR	(95% CI)	*p*
Study Group	No DM		1 (ref.)		
	NOT2DM	0.342 (0.058)	1.408	(1.256; 1.578)	<0.001
	T2DM	0.101 (0.082)	1.106	(0.942; 1.299)	0.218
Age	One-year increase	0.069 (0.003)	1.071	(1.065; 1.077)	<0.001
Sex	Male vs. Female	0.126 (0.060)	1.135	(1.009; 1.276)	0.035
Cardiomegaly		0.209 (0.088)	1.233	(1.037; 1.465)	0.018
Supraventricular arrhythmias		0.258 (0.067)	1.295	(1.135; 1.477)	<0.001
CHF		0.244 (0.076)	1.277	(1.100; 1.483)	0.001
s/p MI		0.197 (0.073)	1.217	(1.054; 1.405)	0.007
Chronic kidney disease		0.501 (0.099)	1.651	(1.360; 2.005)	<0.001
COPD		0.576 (0.088)	1.779	(1.497; 2.115)	<0.001
Neurological disorders		0.340 (0.074)	1.406	(1.216; 1.625)	<0.001
Anemia		0.259 (0.057)	1.296	(1.158; 1.449)	<0.001
Schizophrenia/Psychosis		0.601 (0.185)	1.825	(1.269; 2.624)	0.001
Alcohol/drug addiction		0.651 (0.173)	1.917	(1.366; 2.690)	<0.001
Type of AMI	NSTEMI vs. STEMI	0.177 (0.057)	1.193	(1.068; 1.334)	0.002
Type of treatment	Noninvasive		1 (ref.)		
	PCI	−0.407 (0.067)	0.666	(0.583; 0.760)	<0.001
	CABG	−0.631 (0.101)	0.532	(0.437; 0.649)	<0.001
Severe LV dysfunction		0.414 (0.099)	1.512	(1.245; 1.836)	<0.001
Pulmonary hypertension		0.391 (0.106)	1.479	(1.202; 1.820)	<0.001
Year of admission	One-year increase	−0.002 (0.008)	0.998	(0.983; 1.013)	0.818
Period from discharge to index date	One-year increase	0.094 (0.010)	1.099	(1.078; 1.120)	<0.001

AdjHR: adjusted hazard ratio; AMI: acute myocardial infarction; B (SE): beta coefficient (standard error); CABG: coronary artery bypass grafting; CHF: congestive heart failure; CI: confidence interval; COPD: chronic obstructive pulmonary disease; LV: left ventricular; MI: myocardial infarction; NSTEMI: non-ST-elevation myocardial infarction; PCI: percutaneous coronary intervention; s/p: status post; STEMI: ST-elevation myocardial infarction; T2DM: type 2 diabetes mellitus.

**Table 3:** Relationships between the status of type 2 diabetes mellitus and the risk for post-acute myocardial infarction all-cause mortality throughout the follow-up period up to 10 years, by age strata—multivariable analyses.

Age Strata < 65 Years					
Parameter	Value	B (SE)	AdjHR	(95% CI)	*p*
Study Group	No DM		1 (ref.)		
	NOT2DM	0.611 (0.105)	1.842	(1.500; 2.261)	<0.001
	T2DM	0.383 (0.148)	1.466	(1.098; 1.958)	0.010
Age	One-year increase	0.050 (0.007)	1.051	(1.036; 1.066)	<0.001
Sex	Male vs. Female	0.076 (0.132)	1.079	(0.833; 1.397)	0.564
Cardiomegaly		0.218 (0.174)	1.243	(0.884; 1.748)	0.211
Supraventricular arrhythmias		0.558 (0.158)	1.747	(1.283; 2.380)	<0.001
CHF		0.382 (0.163)	1.465	(1.064; 2.018)	0.019
s/p MI		0.386 (0.138)	1.471	(1.123; 1.928)	0.005
Chronic kidney disease		1.090 (0.229)	2.974	(1.900; 4.657)	<0.001
COPD		0.677 (0.193)	1.968	(1.349; 2.871)	<0.001
Neurological disorders		0.814 (0.147)	2.258	(1.693; 3.010)	<0.001
Anemia		0.198 (0.116)	1.219	(0.972; 1.530)	0.087
Schizophrenia/Psychosis		0.890 (0.316)	2.434	(1.309; 4.526)	0.005
Alcohol/drug addiction		1.144 (0.220)	3.14	(2.041; 4.832)	<0.001
Type of AMI	NSTEMI vs. STEMI	0.154 (0.100)	1.166	(0.959; 1.419)	0.124
Type of treatment	Noninvasive		1 (ref.)		
	PCI	−0.398 (0.144)	0.671	(0.506; 0.891)	0.006
	CABG	−0.630 (0.197)	0.533	(0.362; 0.783)	0.001
Severe LV dysfunction		0.670 (0.175)	1.954	(1.387; 2.752)	<0.001
Pulmonary hypertension		0.616 (0.303)	1.852	(1.022; 3.355)	0.042
Year of admission	One-year increase	0.035 (0.015)	1.036	(1.006; 1.067)	0.019
Period from discharge to index date	One-year increase	0.115 (0.016)	1.121	(1.087; 1.157)	<0.001
**Age strata 65–75 years**					
Study Group	No DM		1 (ref.)		
	NOT2DM	0.219 (0.114)	1.244	(0.995; 1.557)	0.056
	T2DM	0.163 (0.155)	1.177	(0.868; 1.596)	0.294
Age	One-year increase	0.095 (0.018)	1.1	(1.062; 1.14)	<0.001
Sex	Male vs. Female	0.244 (0.114)	1.276	(1.021; 1.594)	0.032
Cardiomegaly		0.277 (0.161)	1.319	(0.962; 1.809)	0.085
Supraventricular arrhythmias		0.401 (0.125)	1.494	(1.169; 1.910)	0.001
CHF		0.188 (0.162)	1.207	(0.879; 1.658)	0.244
s/p MI		0.026 (0.141)	1.027	(0.778; 1.354)	0.853
Chronic kidney disease		0.779 (0.188)	2.179	(1.507; 3.151)	<0.001
COPD		0.609 (0.150)	1.839	(1.371; 2.468)	<0.001
Neurological disorders		0.197 (0.164)	1.218	(0.882; 1.681)	0.230
Anemia		0.470 (0.105)	1.601	(1.302; 1.968)	<0.001
Schizophrenia/Psychosis		0.261 (0.396)	1.298	(0.598; 2.820)	0.51
Alcohol/drug addiction		1.198 (0.356)	3.314	(1.649; 6.660)	<0.001
Type of AMI	NSTEMI vs. STEMI	0.382 (0.106)	1.465	(1.189; 1.804)	<0.001
Type of treatment	Noninvasive		1 (ref.)		
	PCI	−0.482 (0.127)	0.617	(0.481; 0.792)	<0.001
	CABG	−0.940 (0.185)	0.391	(0.272; 0.561)	<0.001
Severe LV dysfunction		0.410 (0.193)	1.507	(1.032; 2.202)	0.034
Pulmonary hypertension		0.586 (0.211)	1.797	(1.189; 2.717)	0.005
Year of admission	One-year increase	−0.017 (0.015)	0.983	(0.955; 1.011)	0.236
Period from discharge to index date	One-year increase	0.080 (0.018)	1.083	(1.045; 1.123)	<0.001
**Age strata 75 years and above**					
Study Group	No DM		1 (ref.)		
	NOT2DM	0.314 (0.094)	1.369	(1.138; 1.647)	<0.001
	T2DM	−0.127 (0.131)	0.881	(0.681; 1.140)	0.334
Age	One-year increase	0.059 (0.009)	1.061	(1.043; 1.080)	<0.001
Sex	Male vs. Female	0.202 (0.086)	1.223	(1.033; 1.448)	0.019
Cardiomegaly		0.075 (0.137)	1.078	(0.825; 1.410)	0.582
Supraventricular arrhythmias		0.107 (0.092)	1.113	(0.930; 1.333)	0.243
CHF		0.213 (0.105)	1.237	(1.007; 1.520)	0.043
s/p MI		0.122 (0.115)	1.129	(0.901; 1.415)	0.291
Chronic kidney disease		0.242 (0.138)	1.274	(0.972; 1.669)	0.079
COPD		0.411 (0.140)	1.508	(1.146; 1.986)	0.003
Neurological disorders		0.237 (0.102)	1.267	(1.037; 1.548)	0.020
Anemia		0.182 (0.086)	1.200	(1.013; 1.422)	0.035
Schizophrenia/Psychosis		0.540 (0.292)	1.716	(0.968; 3.042)	0.065
Alcohol/drug addiction		−0.430 (0.470)	0.651	(0.259; 1.635)	0.361
Type of AMI	NSTEMI vs. STEMI	0.043 (0.093)	1.044	(0.870; 1.253)	0.640
Type of treatment	Noninvasive		1 (ref.)		
	PCI	−0.477 (0.101)	0.621	(0.509; 0.757)	<0.001
	CABG	−0.582 (0.175)	0.559	(0.397; 0.788)	<0.001
Severe LV dysfunction		0.333 (0.160)	1.395	(1.020; 1.908)	0.037
Pulmonary hypertension		0.333 (0.137)	1.396	(1.068; 1.824)	0.015
Year of admission	One-year increase	−0.006 (0.012)	0.994	(0.972; 1.017)	0.604
Period from discharge to index date	One-year increase	0.103 (0.018)	1.108	(1.070; 1.147)	<0.001

AdjHR: adjusted hazard ratio; AMI: acute myocardial infarction; B (SE): beta coefficient (standard error); CABG: coronary artery bypass grafting; CHF: congestive heart failure; CI: confidence interval; COPD: chronic obstructive pulmonary disease; LV: left ventricular; MI: myocardial infarction; NSTEMI: non-ST-elevation myocardial infarction; PCI: percutaneous coronary intervention; s/p: status post; STEMI: ST-elevation myocardial infarction; T2DM: type 2 diabetes mellitus.

## Data Availability

The data underlying this article may be shared upon reasonable request to the corresponding author.
